# GLA-AF, an Emulsion-Free Vaccine Adjuvant for Pandemic Influenza

**DOI:** 10.1371/journal.pone.0088979

**Published:** 2014-02-14

**Authors:** Christopher H. Clegg, Richard Roque, Lucy A. Perrone, Joseph A. Rininger, Richard Bowen, Steven G. Reed

**Affiliations:** 1 TRIA Bioscience Corp, Seattle, Washington, United States of America; 2 CaroGen Corporation, Hamden, Connecticut, United States of America; 3 Colorado State University, Fort Collins, Colorado, United States of America; 4 Infectious Disease Research Institute, Seattle, Washington, United States of America; Centers for Disease Control and Prevention, United States of America

## Abstract

The ongoing threat from Influenza necessitates the development of new vaccine and adjuvant technologies that can maximize vaccine immunogenicity, shorten production cycles, and increase global vaccine supply. Currently, the most successful adjuvants for Influenza vaccines are squalene-based oil-in-water emulsions. These adjuvants enhance seroprotective antibody titers to homologous and heterologous strains of virus, and augment a significant dose sparing activity that could improve vaccine manufacturing capacity. As an alternative to an emulsion, we tested a simple lipid-based aqueous formulation containing a synthetic TLR4 ligand (GLA-AF) for its ability to enhance protection against H5N1 infection. GLA-AF was very effective in adjuvanting recombinant H5 hemagglutinin antigen (rH5) in mice and was as potent as the stable emulsion, SE. Both adjuvants induced similar antibody titers using a sub-microgram dose of rH5, and both conferred complete protection against a highly pathogenic H5N1 challenge. However, GLA-AF was the superior adjuvant in ferrets. GLA-AF stimulated a broader antibody response than SE after both the prime and boost immunization with rH5, and ferrets were better protected against homologous and heterologous strains of H5N1 virus. Thus, GLA-AF is a potent emulsion-free adjuvant that warrants consideration for pandemic influenza vaccine development.

## Introduction

Avian Influenza viruses circulate widely in aquatic birds and five subtypes (H5N1, H7N3, H7N7, H7N9 and H9N2) are known to cause illness in people. While disease symptoms can often be mild, infection by H5N1 and H7N9 can be life-threatening. The first human outbreak of H5N1 was identified in 1997, and since it reemerged in 2003, more than 640 cases have been identified to date with an approximate 60% mortality rate [1]. The recent outbreak of H7N9 in early 2013 infected 131 people within 2 months, 36 of whom died, and evidence indicated that this virus could be transmitted between ferrets and possibly humans [2]–[5]. The discovery that the H7N7 subtype virus can also infect mammals reinforces the belief that pandemic threats extend beyond H5N1 and H7N9 subtypes [2]. To meet this threat, pandemic preparedness goals in the US include improving manufacturing capability to supply the necessary quantities of vaccine within six months of the declaration of a pandemic, and stockpiling pre-pandemic vaccines that might provide complete or partial coverage against a new virus. To achieve these goals, new recombinant technologies and adjuvants are needed that will shorten production cycles, maximize vaccine immunogenicity, and increase global vaccine supply.

Effective adjuvants can augment protective immune responses using minimal antigen, thus providing a dose-sparing benefit that increases vaccine coverage. The most widely used class of adjuvants for Influenza vaccines are oil-in-water emulsions including MF59, ASO3, and AF03. These adjuvants contain shark-derived squalene that is microfluidized in buffer and surfactants to generate oil particles, averaging 100–160 nm in diameter, suspended in water [6]–[8]. Collectively, these emulsions induce seroprotective antibody responses to inactivated H5N1 vaccines that exceed approvable endpoint criteria in humans and provide a significant dose sparing effect [6]–[11]. In addition, they mediate the priming and production of cross-reactive H5N1 antibody responses that recognize drifted strains of virus. Despite the fact these emulsions are approved for human use in the European Union, there has not been a product registration that includes emulsion-based adjuvants in the US [12], [13].

Another type of adjuvant targets Toll-like receptors (TLRs) on antigen presenting cells and the induction of Th1-mediated immune responses (14). TLR4 agonists are highly effective in experimental and clinical settings with the most advanced product being MPL (monophosphoryl lipid A), a component of the human papilloma virus vaccine, Cervarix®, which received FDA approval in October of 2009 [15]. We recently described the activity of an H5N1 vaccine containing a recombinant HA (rH5) protein and GLA-SE, a two part adjuvant formulation using the synthetic TLR4 agonist Glucopyranosyl Lipid Adjuvant (GLA) and a stable oil-in-water emulsion. It was determined that a single submicrogram dose of adjuvanted vaccine protected mice and ferrets against a high titer challenge with a H5N1 virus, and that GLA-SE, relative to emulsion alone, broadened protective immunity against heterosubtypic viruses [16]. Notwithstanding these results, we have continued to test simpler GLA adjuvant formulations that lack animal products and are easier to produce. Here we show that an aqueous GLA formulation (GLA-AF) containing 100 nM particles and the synthetic surfactant DPPC (dipalmitoylphosphatidylcholine) [17], is a very potent adjuvant for rH5 vaccines in mice and ferrets and represents an important alternative to oil-in-water emulsions for pandemic Influenza vaccines.

## Materials and Methods

### Ethics Statement

This study was carried out in strict accordance with the recommendations in the Guide for the Care and Use of Laboratory Animals of the National Institutes of Health, the US Public Health Service Policy on Humane Care and Use of Laboratory Animals, and the Association for Assessment and Accreditation of Laboratory Animal Care International (AAALAC). Protocol #2012-9 was approved by the Institutional Animal Care and Use Committees of the Infectious Disease Research Institute which operates under a currently approved Assurance #A4337-01 and USDA certificate #91-R-0061. Experimental protocol #11-2417 was approved by the Institutional Animal Care and Use Committees of Colorado State University (Assurance #A7248-54). Animal welfare and health was monitored daily and in instances where medical intervention was not effective, animals were humanely euthanized and every effort was made to minimize suffering.

### Animals and Viruses

Female C57BL/6 mice (Jackson Labs, Bar Harbor, ME) were maintained under pathogen-free conditions and all experimental protocols were conducted in accordance with the guidelines of the Infectious Disease Research Institute (IDRI, Seattle, WA) and Colorado State University Animal Care and Use Committees. Male fitch ferrets (Triple F Farms, Sayre, PA) were maintained at The Regional Biocontainment Laboratory, Colorado State University (Fort Collins, CO). The H5N1 virus isolate A/Vietnam/1203/2004 (Clade 1) was obtained from the Influenza Division at the Centers for Disease Control and Prevention (Atlanta, GA). Virus stocks were prepared by inoculation of 10-day old embryonated chicken eggs (Sunrise Farms, Catskill, NY) and titrated by plaque assay on MDCK cells using standard methods.

### Immunization and Viral Challenge

Recombinant H5N1 Hemagglutinin protein (rH5; Protein Sciences Corporation, Meriden, CT) was combined on the day of immunization with the following adjuvants: phosphate buffered saline (PBS), SE (final 2% oil-in-water stable emulsion), or GLA-AF containing 1 µg, 5 µg, or 20 µg of GLA. Mice were injected with 50 µL in each hind quadriceps muscle (100 µL total volume) and ferrets were injected with 250 µL vaccine in the left quadriceps muscle. Experiments using the highly pathogenic H5N1 influenza virus were performed under the guidance of the U.S. National Select Agent Program in negative pressure HEPA-filtered BSL-3 laboratories at Colorado State University. Virus challenge was carried out using intra-nasal inoculation with virus diluted in sterile PBS. Mice received the indicated virus dose in a total volume of 25 µL split into each nare. Ferret inoculations were carried out in a total volume of 1 mL, with 500 µL inoculated into each nare. Following virus challenge, animals were monitored for weight loss, body temperature (ferret), and general health using a 5 point clinical score (0 = normal to 4 = poorly responsive or moribund). Animals were euthanized humanely if body weight loss reached 25% or clinical appearance was scored as 4.

### Serology

Hemagglutination Inhibition (HI) activity specific to H5N1 virus antigens used in this study (National Institute for Biological Standards and Control, Hertfordshire, England) was determined using 1% horse red blood cells as previously described [18]. Individual serum samples were treated with receptor-destroying enzyme (RDE) (Denka-Seiken, Tokyo, Japan) overnight followed by heat-inactivation to remove non-specific inhibitors. HI titer is defined as the reciprocal of the highest dilution of sera which completely inhibits the agglutination of the horse RBCs. The limit of detection for this assay is a 1∶10 dilution. HI antibody responses against the vaccine strain of rH5 Indo were not measured because of the unavailability of the inactivated virus. Total IgG, IgG1, and IgG2c responses were measured by ELISA using standard methods. Briefly, 96-well plates were coated overnight with rH5 protein (1 µg/ml) and incubated with serum dilutions for 1 hr, rinsed, and bound immunoglobulin was detected using secondary anti-mouse IgG reagents. Assay results were expressed using end-point titration values. The limit of detection for this assay was a 1∶1000 dilution.

### T cell Assays

Antigen-specific T cell responses were measured following rH5 protein stimulation *in vitro*. Briefly, splenocytes were isolated from immunized mice and cultured in complete media (4×10^6^ cells/well) with 1 µg/ml rHA A/Vietnam/1203/2004 protein. Forty eight hours later, cytokines (IL-2, IL-3, IL-4, IL-5, IL-6, IL-10, GM-CSF, IFN-g, and TNF-a) were measured from supernatants using Bio-Plex Pro™ mouse cytokine Th1/Th2 immunoassay, a Luminex xMAP® multiplex technology (Bio-Rad, Hercules, CA), and analyzed on a Bio-Plex®200 System (Bio-Rad) running Bio-Plex Manager™ Software (Bio-Rad). The negative and positive controls for these assays were splenocytes cultured in medium alone, non-H5 peptides, and anti-CD3+ anti-CD28 mAb.

### Statistical Analysis

Comparisons between vaccinated groups were performed using a non-parametric one-way ANOVA with the Turkey Multiple Comparison Test and a Fisher’s exact test. The analyses were performed using GraphPad Prism version 5.0 for Windows (GraphPad Software, San Diego, CA). P values of less than 0.05 were considered to be significant.

## Results

### GLA-AF Induces Protective Th1-mediated Antibody Responses in Mice

**Figure 1:**
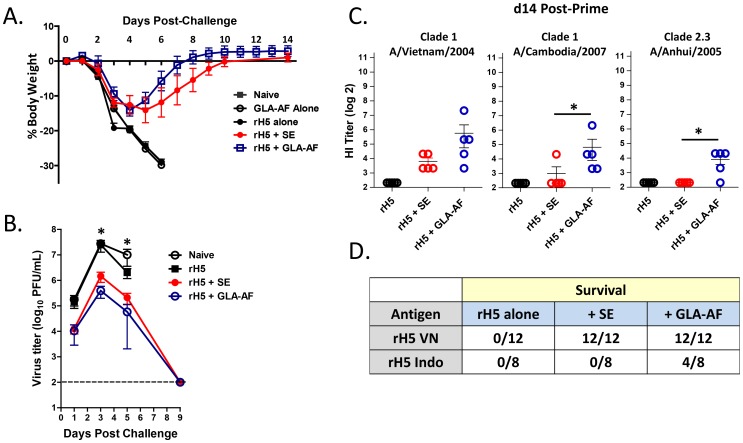
GLA-AF augments priming of a protective immune response. Mice (5/group) received a prime immunization on d0 with rH5 VN (50 ng) either alone, with SE, or with GLA-AF (1 µg), and were then challenged 14 days later with 1000 LD50 A/H5N1/Vietnam/1203/04. (A) Mean percent change in body weight (+/− SEM) post-challenge (representative data from three experiments). (B) Kinetics of virus clearance. Each point is the average virus titer measured in mouse lung homogenate (n = 3), where 1 ml represents approximately 20% of lung volume. Statistical differences (p<0.05) between the adjuvanted and non-adjuvanted groups on day 3 and day 5 are indicated by the asterisks. (C) Day 14 sera were assayed for HI titers directed against the clade 1 vaccine HA or 2 heterosubtypic HA antigens. Each bar presents the geometric mean titer and statistical differences (p<0.001, Fisher’s test) between SE and GLA-AF are indicated. (D) Cumulative survival of mice immunized once with 50 ng of rH5 VN or rH5 Indo antigen and then challenged 14 days later with 1000 LD50 A/H5N1/Vietnam/1203/04.

**Figure 2:**
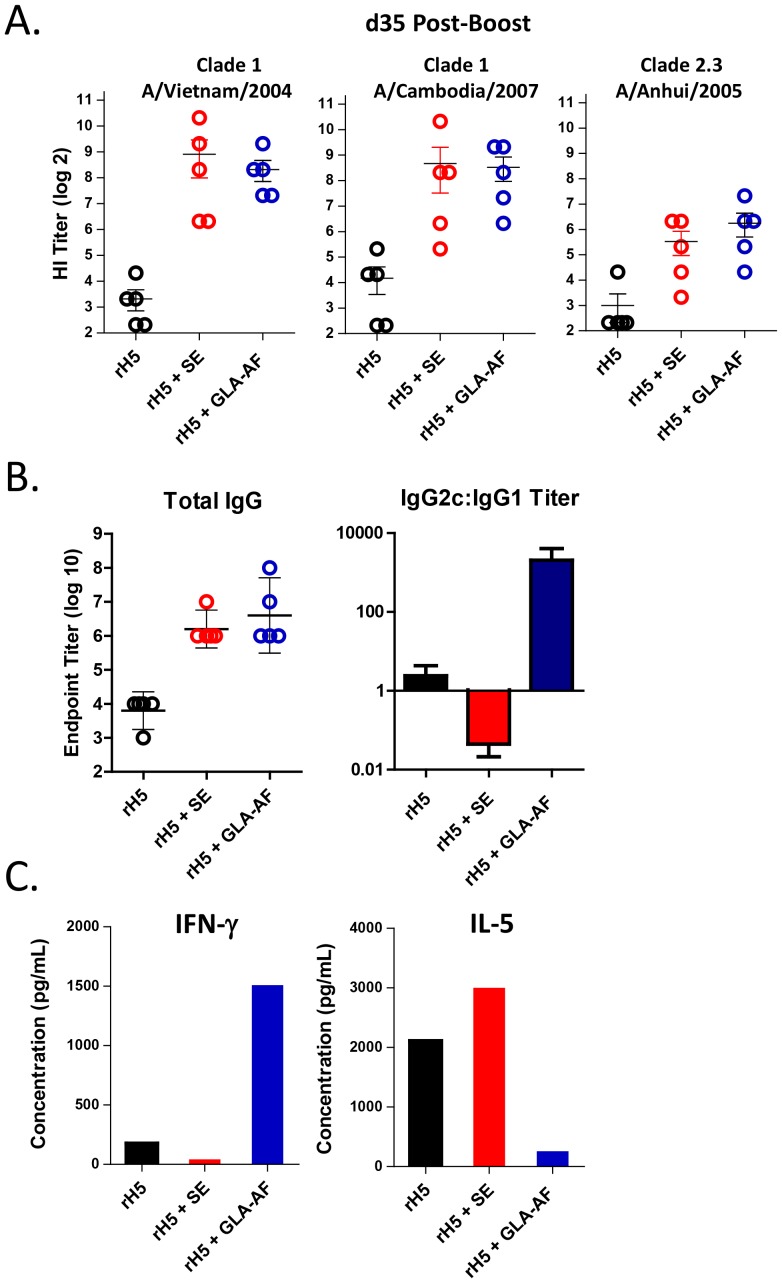
GLA-AF stimulates a Th1 antibody response. Mice (5/group) received a prime (d0)+boost (d21) immunization with rH5VN (50 ng) ± adjuvant. (A) HI titers directed against the homologous vaccine strain of virus and 2 drifted virus. (B) Total anti-H5 IgG titers measured by ELISA and the ratio of IgG2c and IgG1 isotype titers. (C) Relative production of IL-5 and IFN-γ in antigen stimulated splenocytes as measured by Luminex.

We first compared the relative potency of GLA-AF to the squalene-based oil-in-water emulsion (SE) using an assay that measures adjuvant-dependent priming of a protective immune response [16]. C57BL/6 mice were immunized once with 50 ng of recombinant HA from A/Vietnam/1203/04 (rH5VN), either alone or with GLA-AF and SE adjuvants, and then challenged 14 days later with 1000 LD50 A/Vietnam/1203/04 virus. As indicated in Figure 1A, all mice lost weight after viral challenge. Naïve mice and animals injected with either antigen or adjuvant alone died within six days, while 100% of mice injected with rH5VN plus GLA-AF or SE regained weight and survived. Typically, mice immunized with H5+ GLA-AF appeared to recover body weight faster than the H5+ SE groups, although no difference in viral clearance was seen (Figure 1B). These mice also tended to have a greater HI antibody response directed against the homologous vaccine antigen (Figure 1C), but again, survival was the same (100%) in both adjuvant groups. However, GLA-AF was more effective than SE in stimulating cross-reactive antibodies to heterosubtypic H5N1 antigens (Figure 1C) and could partially protect mice when immunized with rH5 derived from A/Indonesia/05/05 (rH5 Indo) and then challenged with the heterosubtypic A/Vietnam/1203/04 virus (Figure 1D). These results demonstrate that GLA-AF is a potent adjuvant for stimulating a protective immune response following a single vaccination. We next investigated the effects of GLA-AF in a prime-boost immunization and challenge experiment. Unlike the prime-only experiment, we observed no significant differences in the ability of GLA-AF or SE to enhance rH5-mediated antibody responses to homologous and heterologous H5 antigen (Figure 2A), and 100% of mice in both adjuvant groups survived a challenge with a heterologous virus (data not shown). Previously, we reported that GLA-SE enhances antiviral protection through a Th1-mediated immune response [16] and we tested whether GLA-AF might work by a similar mechanism. A measurement of circulating antibodies by ELISA indicated that while the total antigen-specific IgG was the same between groups, GLA-AF induced anti-H5 IgG2c preferentially, whereas SE stimulated production of IgG1 (Figure 2B). In addition, when splenocytes from GLA-AF immunized mice were stimulated with H5 protein *in vitro*, they expressed IFN-γ and very little IL-5, whereas cells isolated from the SE group produced primarily IL-5. Collectively, these results show that GLA-AF induces Th1-mediated immune responses and is very effective in protecting mice against high titers challenges with H5N1 virus.

### GLA-AF is Superior to SE in Stimulating Protective Immunity in Ferrets

**Figure 3:**
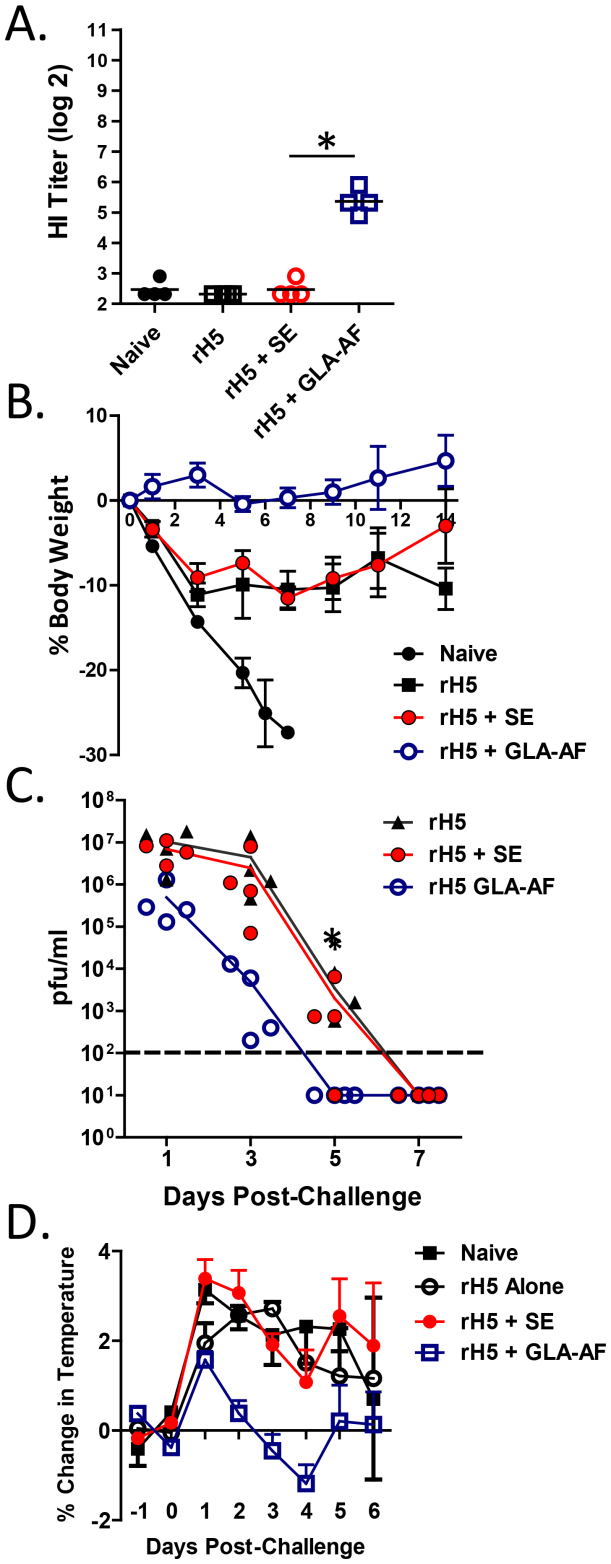
GLA-AF augments priming of protective immune responses in ferrets. Animals (4/group) injected once with rH5 VN (0.5 µg) alone or with adjuvant (SE, 20 µg GLA-AF) were challenged on d28 with 7.5×10^5^ pfu A/Vietnam/1203/04. Survival per immunization group was: naïve (0/4), rH5 alone (2/4), rH5+ SE (4/4), rH5+ GLA-AF (4/4). (A) Serum HI titers in d28 sera. Each bar presents the geometric mean titer and statistical difference (p<0.001) between SE and GLA-AF is indicated by the asterisk. (B) Mean percent change in body weight (+/− SEM). (C) Daily changes in viral load measured in nasal washes. The asterisk on day 5 denotes a significant difference in detectable virus between vaccine groups adjuvanted with GLA-AF and SE (p = 0.028; Fisher’s exact test). (D) Mean percent change in body temperature (+/− SEM).

**Figure 4:**
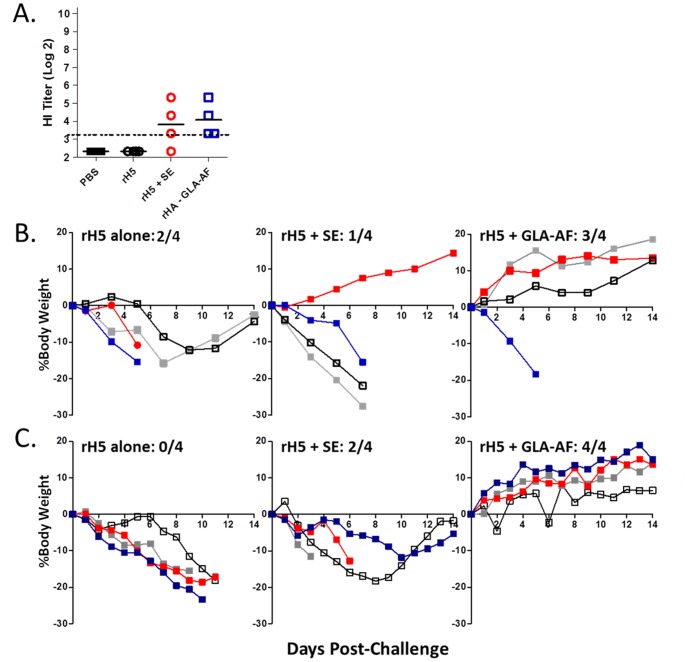
GLA-AF enhances protective priming against a heterosubtypic H5N1 virus in ferrets. Animals (4/grp) injected with rH5 Indo (0.5 µg) alone or with adjuvant (SE, 20 µg GLA-AF) were challenged on d28 with 7.5×10^5^ pfu A/Vietnam/1203/04. (A) HAI titers directed against the heterologous challenge strain, A/Vietnam/1203/04. (B) Survival and the percent change in body weights per immunization group. (C) Animals (4/grp) injected with rH5 Indo (300 ng) alone or with adjuvant (SE, 20 µg GLA-AF) were challenged on d14 with 7.5×10^5^ pfu A/Vietnam/1203/04.

The GLA-formulated emulsion, GLA-SE, accelerates and broadens induction of a primary protective immune response in ferrets relative to an emulsion alone [16]. To test GLA-AF in a similar fashion, we injected animals once with 0.5 µg of rH5 VN +/− adjuvant and challenged 28 days later with the homologous A/Vietnam/1203/04 virus (Figure 3). As indicated, sera collected at the time of challenge contained detectable HI titers in only the GLA-AF group. After challenge, all four animals in the GLA-AF and SE groups survived, while 2 of 4 animals in the antigen alone group and 0 out of 4 in the naïve group survived. Animals receiving rH5+ GLA-AF demonstrated improved overall health as compared to the other groups as measured by changes in body weight and body temperature. Importantly, lung viral load was significantly reduced and infection cleared more rapidly in the GLA-AF groups. GLA-AF was also superior to SE in two heterologous challenge experiments. In the first (Figure 4), ferrets received one injection of 0.5 µg rH5 Indo followed by challenge on day 28 with A/H5N1/Vietnam/1203/04. Survival in this experiment was the following; naïve (0/4), rH5 alone (2/4), rH5+ SE (1/4), and rH5+ GLA-AF (3/4). GLA-AF and SE induced similar low titers of cross-reactive antibodies against the challenge virus (Figure 4A), although animals were better protected with GLA-AF than SE in this assay (Figure 4B). In a second more stringent test (Figure 4C), animals received less antigen (0.3 µg rH5 Indo) and were challenged just 14 days after the immunization. Survival in this experiment was: naïve (0/4), rH5 Indo alone (0/4), rH5+ SE (2/4), and rH5+ GLA-AF (4/4). While antibody responses were undetectable prior to challenge, GLA-AF improved the ability to maintain normal weight in the surviving animals. These results demonstrate that GLA-AF is more effective than SE in stimulating single dose protection against homologous and heterologous H5N1 viruses in ferrets.

**Figure 5:**
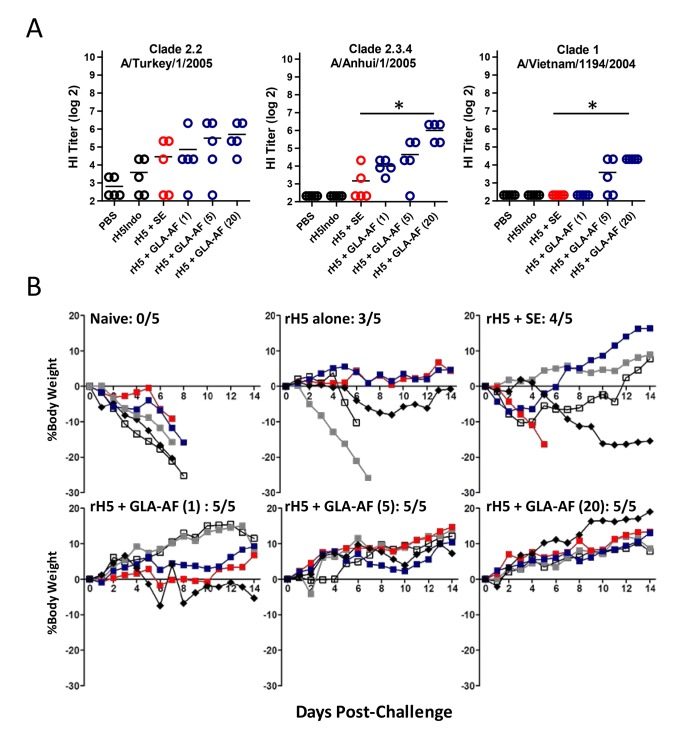
GLA-AF enhances protection against a heterosubtypic H5N1 virus in ferrets. Animals (5/group) received a prime (d0)+boost (d21) immunization with rH5 Indo (2 µg) either alone, with SE, or with 1 µg, 5 µg, and 20 µg GLA-AF. (A) HI titers directed against the indicated strains of virus were assayed in d35 sera. Each bar presents the geometric mean titer and statistical differences (p<0.001, student’s t-test) between SE and 20 µg GLA-AF are indicated by the asterisks. (B) Survival and the percent change in body weights in animals challenged on d42 with 7.5×10^5^ pfu A/Vietnam/1203/04. All of the ferrets in this experiment had detectable virus titer in nasal washes one day after the challenge. No statistical difference in viral clearance between the GLA-AF and SE groups was observed (data not shown).

Emulsion-based adjuvants mediate more than a 30-fold dose sparing effect in humans immunized with inactivated H5N1 vaccines and stimulate seroprotective antibody responses with as little as 1.9–3.8 µg of HA antigen (11). Thus, to test GLA-AF using a regimen that might be used in the clinic, ferrets (5/group) received a prime-boost immunization with 2 µg of H5 Indo either alone, with SE, or increasing doses of GLA-AF (1 µg, 5 µg, and 20 µg). PBS was used as a negative control. Twenty one days after the boost injection, animals were challenged with 7.5×10^5^ pfu of the heterologous A/Vietnam/1203/04 virus and then monitored for weight loss and survival. After measuring HI titers in sera collected on the day of challenge (Figure 5A), we observed that GLA-AF induced a broader antibody response to 3 heterosubtypic viruses compared with SE. In addition, all of the GLA-AF immunized ferrets (15/15) survived the heterologous challenge and 14 of these showed consistent weight gain and appeared completely normal over the course of the experiment (Figure 5B). Within the SE group, one animal died and three lost varying degrees of weight and were scored as having mild illness during the 14 day observation period. Three out of five animals in the rH5 alone group survived, 2 of which were scored as having mild and moderate illness. These data show that prime-boost immunization of ferrets with a clinically relevant dose of rH5+ GLA-AF stimulates a robust protective response against heterologous H5N1 infection.

## Discussion

Vaccine adjuvants are needed for increasing the breadth of protective immunity against Influenza viruses and for improving vaccine manufacturing capacity. Collectively, adjuvants stimulate innate immune pathways that facilitate dendritic cell activation and antigen presentation. Oil-in-water emulsions are very effective adjuvants for pandemic influenza vaccines and stimulate approvable HI titers in adults, young children and infants at substantially reduced doses of antigen [6]–[11]. These adjuvants are prepared by mixing shark-derived squalene and egg phosphatidylcholine oils with an aqueous buffered solution containing glycerol and the surfactant poloxamer 188, followed by high pressure homogenization and filtering [19]. Mechanistically, emulsions induce a chemokine-driven gradient at the site of injection that recruits leukocyte infiltration and antigen transport to local lymph nodes, and their induction of broadly reactive CD4 T cells predict the rise of neutralizing antibody titers after booster immunizations [20], [21]. The humoral responses induced by these adjuvants involve antibody epitope spreading from the HA2 portion of HA to large conformational domains in the globular HA1 region of HA, which correlate with broad cross-clade neutralization [22]. Despite their utility, vaccines containing emulsion-based adjuvants have not been approved in the United States, and ongoing large-scale epidemiological studies are evaluating their safety [23]–[26].

Another important class of adjuvants exemplified by MPL targets TLR4 receptors on DC and the priming of antiviral Th1 CD4 T cells [14]. These cells can inhibit viral replication via production of IFNα and TNFα, they can be directly cytotoxic and regulate CD8 T cell-mediated cell killing, and they are critically important for antiviral antibody responses [27]–[29]. MPL is a detoxified bacterial lipopolysaccharide processed from *Salmonella minnesota*. As a natural product, MPL contains a heterogeneous mix of hexa-, penta-, and tetra-acylated lipid A moieties, only one of which (the hexa-acylated form) was shown to bind human TLR4 efficiently in a similar product [30], [31]. GLA adjuvants contain a synthetic TLR4 agonist that is a pure hexa-acylated molecule with a higher specific activity for human TLR4 than MPL and is potent in stimulating human DC activation and cytokine production *in vitro* [32]. Like MPL, GLA can be formulated with other adjuvants such as alum and oil-in-water emulsions, or formulated alone with liposomes and surfactants [33].

In an effort to develop a simple adjuvant system for pandemic influenza, we compared the relative potencies of GLA-AF, an aqueous formulation of GLA and DPPC, and the emulsion-based adjuvant SE in two preclinical animal models. In mice, each adjuvant induced complete antigen-dependent protection within 14 days following a single immunization, although GLA-AF showed slightly improved d14 HI titers and recovery from weight loss, stimulated a better antibody response to drifted viruses, and partially protected mice against a heterologous virus (Figure 1). Similar to our findings with GLA-SE, which stimulates a protective Th1-mediated antibody response, mice immunized with GLA-AF produced IgG2c antibodies and IFN-γ (Figure 2), whereas SE induced a Th2 response [16]. Following a prime/boost immunization, no significant differences in antibody responses were detected between these adjuvants (Figure 2) and both mediated complete protection against a heterologous virus (data not shown).

The suggestion that GLA-AF accelerates induction of a primary protective immune response relative to an emulsion was confirmed in ferrets, which are the preferred animal model for influenza vaccine development [34]. GLA-AF stimulated a better antibody response after a single immunization and a faster recovery from infection as reflected in measures of body weight and temperature and viral load (Figure 3). This difference was even more pronounced in two heterologous challenge studies, where ferrets immunized once with rH5+ GLA-AF demonstrated less morbidity and much better survival than rH5+ SE (Figure 4). GLA-AF was also more effective following a prime/boost immunization with 2 µg rH5. GLA-AF stimulated a broader antibody response than SE, and all animals in this group survived and showed positive weight gain following challenge with a heterologous virus (Figure 5).

In summary, GLA-AF can protect ferrets following prime-boost immunization with a low clinical dose of antigen, as well as stimulate survival following a single injection with a sub-microgram amount of rH5. This suggests that GLA-AF might be an important tool for preventing the rapid spread of a highly pathogenic pandemic virus. Separately, we have compared GLA-AF and GLA-SE activity in our rH5 immunization studies, and while GLA-SE is generally superior to GLA-AF in mice, they are equally effective in stimulating antibody responses and protecting ferrets against a heterologous virus challenge. Importantly, the safety of GLA-AF and GLA-SE has been established in multiple nonclinical safety studies and both have been tested successfully in humans. We conclude that GLA-AF is a potent emulsion-free adjuvant that warrants consideration for pandemic influenza vaccine development.
